# Ivabradine is as effective as metoprolol in the prevention of ventricular arrhythmias in acute non-reperfused myocardial infarction in the rat

**DOI:** 10.1038/s41598-020-71706-3

**Published:** 2020-09-14

**Authors:** Mariusz Marciszek, Aleksandra Paterek, Marta Oknińska, Urszula Mackiewicz, Michał Mączewski

**Affiliations:** Department of Clinical Physiology, Centre of Postgraduate Medical Education, Warsaw, Poland

**Keywords:** Cardiovascular biology, Cardiovascular biology

## Abstract

Ventricular arrhythmias are a major source of early mortality in acute myocardial infarction (MI) and remain a major therapeutic challenge. Thus we investigated effects of ivabradine, a presumably specific bradycardic agent versus metoprolol, a β-blocker, at doses offering the same heart rate (HR) reduction, on ventricular arrhythmias in the acute non-reperfused MI in the rat. Immediately after MI induction a single dose of ivabradine/ metoprolol was given. ECG was continuously recorded and ventricular arrhythmias were analyzed. After 6 h epicardial monophasic action potentials (MAPs) were recorded and cardiomyocyte Ca^2+^ handling was assessed. Both ivabradine and metoprolol reduced HR by 17% and arrhythmic mortality (14% and 19%, respectively, versus 33% in MI, *p* < 0.05) and ventricular arrhythmias in post-MI rats. Both drugs reduced QTc prolongation and decreased sensitivity of ryanodine receptors in isolated cardiomyocytes, but otherwise had no effect on Ca^2+^ handling, velocity of conduction or repolarization. We did not find any effects of potential *I*_Kr_ inhibition by ivabradine in this setting. Thus Ivabradine is an equally effective antiarrhythmic agent as metoprolol in early MI in the rat. It could be potentially tested as an alternative antiarrhythmic agent in acute MI when β-blockers are contraindicated.

## Introduction

Ventricular arrhythmias are a major source of early mortality in acute myocardial infarction (MI)^1^ and remain a major therapeutic challenge, especially in the pre-hospital phase. They are usually of re-entrant nature and include ventricular tachycardia and fibrillation.

β-blockers reduce both ventricular arrhythmias and mortality in acute MI^2^, though mechanism of this effect remains unknown. One possible pathway involves heart rate (HR) reduction and its associated benefits. Indeed elevated HR is a risk factor of post-MI arrhythmias and mortality. Furthermore, the magnitude of arrhythmia reduction by β-blockers is related to HR reduction^3^.

In this context we have recently shown that ivabradine, a putative selective inhibitor of cardiac pacemaker F current (*I*_F_) flowing through hyperpolarization-activated cyclic nucleotide-gated (HCN) channel and pure HR reducing agent that is devoid of hemodynamic effects of β-blockers^4^, reduces ventricular arrhythmias and mortality in the model of acute non-reperfused MI in the rat^5^. However, as it was given as prophylaxis before MI induction and was found to e.g. prevent HCN4 overexpression, it is unknown if its acute administration after MI induction would be equally effective. Moreover, ventricular arrhythmias are induced within minutes of MI induction both in humans and in animal models^6^. Therefore it is unknown if ivabradine administration after MI induction can be effective, especially in the early phase. Furthermore mechanisms of potential antiarrhythmic effects of HR reduction remain unknown. Broadly speaking there are two possibilities: (1) anti-ischemic effects and (2) direct effects of HR reduction on cellular electrophysiology. Therefore, the first aim of our study was to resolve these uncertainties and thus we compared potential anti-arrhythmic effects of an established β-blocker, metoprolol^7^, and ivabradine, at doses providing equal HR reduction, given immediately after induction of a non-reperfused MI in the rat and studied potential mechanisms of these drugs.

Ivabradine is a presumably specific bradycardic agent used to HR rate in the treatment of coronary artery disease and heart failure acting by blocking *I*_F_ current. However recent data indicates that ivabradine also inhibits the rapidly activating delayed rectifier potassium current (*I*_Kr_)^8^. This current is critical for cardiac action potential repolarization and is encoded by the human ether-a-go-go-related gene (hERG). hERG mutations or blockage by drugs resulting in reduction of *I*_Kr_ are principal causes of long QT syndrome predisposing individuals to ventricular arrhythmias and sudden cardiac death^9^. This raises concerns over possible proarrhythmic effects of ivabradine through QT prolongation and increased propensity to ventricular tachycardia (of torsades de poitnes type), in particular in cases when cardiac repolarization reserve is reduced. This might be the case in early MI, since hypoxia was shown to downregulate hERG through upregulation of calpain mediated cleavage^10^ and we indeed demonstrated that hEGR abundance is reduced in post-MI rat hearts as early as 24 h after MI induction^5^.

A recent study in isolated perfused rabbit hearts revealed that in the context of veratridine- or sotalol-induced QT prolongation and increased spatial dispersion of repolarization, ivabradine resulted in further QT prolongation and increased incidence of ventricular tachycardia^11^ and a case report suggested that concomitant administration of ivabradine and azithromycin, a drug known to prolong QT interval, resulted in QT prolongation and induction of torsade de pointes^12^. On the other hand a recent meta-analysis demonstrated that ivabradine increased the risk of atrial fibrillation (AF) in humans enrolled in clinical trials^13^. The mechanism of this effect is currently unknown; may be related to bradycardia that is known to facilitate AF by increasing the dispersion of atrial repolarization. To complicate the issue even more, experimental studies suggest that ivabradine prevents rather than induces AF^14^.

Therefore, the second aim of our study was to study potential proarrhythmic signals and mechanisms of ivabradine versus metoprolol (that offered the same HR reduction, but did not block hERG) in the rat model of acute non-reperfused MI at various levels.

## Results

### Infarct size, heart rate, mortality and arrhythmias

**Figure 1:**
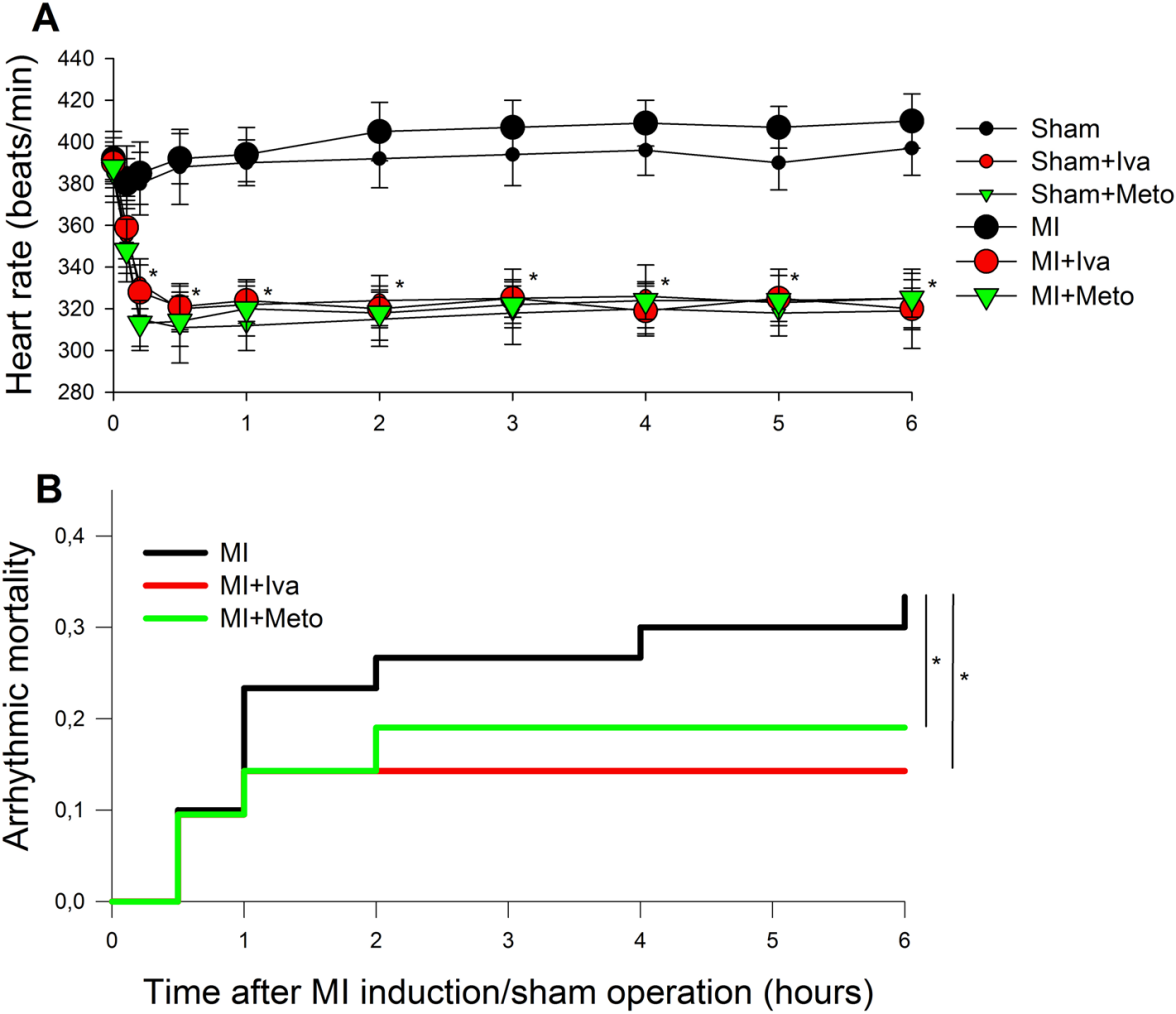
Heart rate profile and arrhythmic mortality within 6 h of myocardial infarction induction or sham operation in rats (**A**) Average heart rate after induction of myocardial infarction (MI) or sham operation (Sham) in saline, ivabradine (Iva) or metoprolol (Meto)-treated rats; (**B**) Kaplan–Meier presentation of arrhythmic mortality. Data are means ± SEM. *p < 0.05 versus respective saline group. For group sizes see Table 1. Figure created using SigmaPlot v.14.0 (https://systatsoftware.com/products/sigmaplot/).

Mean ischemic area estimated by echocardiography did not differ between MI, MI and ivabradine (MI + Iva) or MI and metoprolol (MI + Meto) groups 10 min after coronary artery ligation (Table I), corresponding to eventually ~ 33% infarct size of the LV myocardium. Neither time to peak ST segment elevation in ECG, a marker of the rate of ischemia development, not time to Q wave development in ECG, a marker of the rate of necrosis development, differed between the MI groups (Table I). Average HR over 6 h after sham operation was 390 ± 5, 328 ± 11 and 319 ± 12 bpm and after MI was 399 ± 11, 326 ± 12 and 323 ± 10 bpm, respectively, in saline, Iva and Meto treated rats (both Iva and Meto groups differed significantly from their respective saline-treated Sham and MI controls, *p* < 0,05, while there was no difference between Iva and Meto either in Sham or MI rats) (Fig. 1A).

Within 1 h of MI induction 7, 7 and 8 animals died in MI, MI + Iva and MI + Meto groups, respectively, as well as one animal in the Sham + Meto group, due to bradycardia associated with complete atrioventricular block, without preceding arrhythmia. These deaths were presumed to be caused by surgical complications/cardiogenic shock and were not included in the analysis of arrhythmic mortality. This non-arrhythmic mortality did not differ between the study groups.

Arrhythmic mortality within 6 h after MI was highest in the MI group (33%); both ivabradine (14%) and metoprolol (19%) reduced it significantly, and the difference between these two interventions was not significant. What is interesting, as presented by Kaplan–Meier curves on Fig. 1B, the curves for specific study groups started to diverge only after first 30 min of ischemia. Total mortality did not differ between the study groups (17/37, 10/28, 12/29 in MI, MI + Iva and MI + Meto groups, respectively).

**Figure 2:**
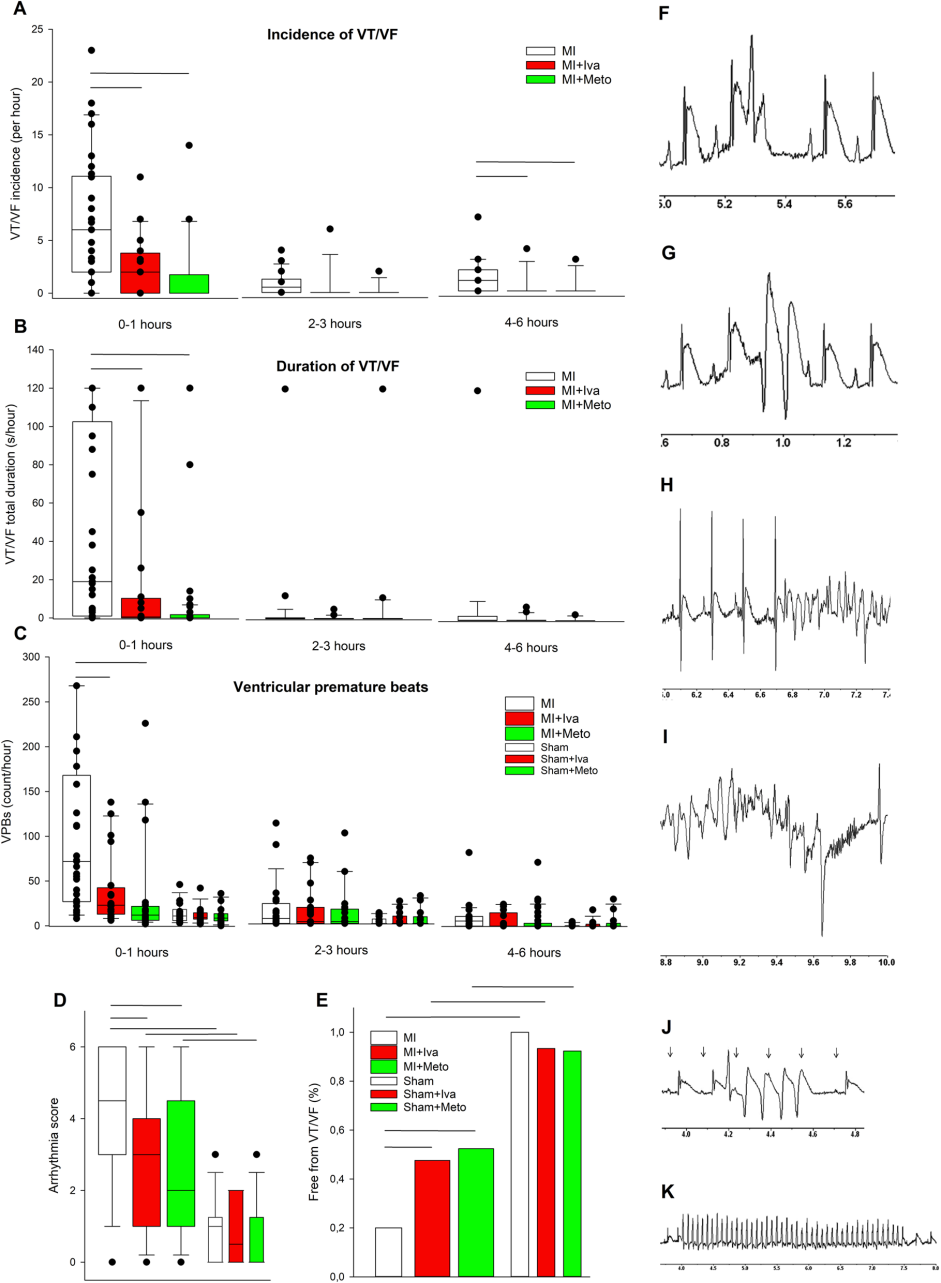
Ventricular arrhythmias within 6 h of myocardial infarction induction or sham operation in rats (**A**) Incidence, (**B**) total duration of ventricular tachycardia/ventricular fibrillation (VT/VF) and (**C**) ventricular premature beat (VPB) count in three periods after MI/sham surgery: 0–1, 2–3 and 4–6 h. Panels A and B do not include data for sham, since only two VT episodes occurred in all sham groups throughout the experiment (in Sham + Iva and Sham + Meto groups). (**D**) Overall arrhythmia score and (**E**) percentage of animals free from VT/VF. Horizontal bars represent median, the bottom and the top of the boxes represent the upper and the lower quartile, he whiskers represent 10th and 90th percentile, while the solid circles represent the individual data points. The horizontal lines show statistical significance, *p* < 0.05. For group sizes see Table 1. (**F**)–(**K**) are sample ECG recordings demonstrating various ventricular arrhythmias in the post-myocardial infarction rats; horizontal axis indicates time in seconds. (**F**) A single early VPB, occurring before the completion of cardiac repolarization. (**G**) A pair of late VPB, occurring after the completion of cardiac repolarization. (**H**) An initiation of polymorphic ventricular tachycardia (VT). (**I**) A spontaneous termination of polymorphic VT. (**J**) A short bout of monomorphic VT (4 beats), initiated by an early VPB; arrows indicate undisturbed sinus P waves. (**K**) A 3.5 s episode of monomorphic VT (approximately 800 bpm). Figure created using SigmaPlot v.14.0 (https://systatsoftware.com/products/sigmaplot/).

As Fig. 2A,B present, both incidence and duration of ventricular arrhythmias, ventricular tachycardia/ventricular fibrillation (VT/VF), were significantly higher in saline-treated MI versus MI + Iva and MI-Meto, while the two latter groups did not differ between themselves. Clearly the intensity of arrhythmias was highest within 1 h after MI induction. This was followed by relatively low-arrhythmic period and a trend toward increased arrhythmia incidence after 4 h of ischemia (Fig. 2A). Of note, there was a significant reduction of VT/VF incidence within 0–1 and 4–6 h after MI induction by both ivabradine and metoprolol. Only two VT episodes occurred in all sham groups throughout the experiment (one episode in Sham + Iva and Sham + Meto groups).

**Table 1:** Morphological characterization of the study groups.

Parameter	Sham (n = 14)	Sham + Iva (n = 15)	Sham + Meto (n = 14	MI (n = 37)	MI + Iva (n = 28)	MI + Meto (n = 29)
Body weight (g)	322 ± 22	335 ± 27	340 ± 29	325 ± 20	331 ± 26	320 ± 28
WMI	22.9 ± 0.0	22.9 ± 0.1	23.0 ± 0.0	16.8 ± 1.3*	16.6 ± 1.5*	16.7 ± 1.6*
Mean time to peak ST segment elevation (min)	–	–	–	102 ± 20	107 ± 25	95 ± 26
Mean time to Q wave development (h)	–	–	–	12.1 ± 1.8	11.8 ± 2.0	13.0 ± 2.1

WMI, wall motion index assessed 10 min after coronary artery ligation; *p < 0.05 vs. corresponding sham.

The incidence of ventricular premature beats (VPBs) was highest during the first hour after induction of MI; ivabradine and metoprolol markedly reduced MI-induced VPBs (Fig. 2C). The combined arrhythmia score (median of scores for individual animals ranging from 0 to 6), a measure of the most severe ventricular arrhythmia, was 4.5 in saline-treated MI rats and was similarly reduced by both ivabradine (3) and metoprolol (2) (Fig. 2D). Only 20% of animals were VT/VF free in saline-treated MI group, while 48% and 52% were VT-VF free in MI + Iva and MI + Meto, respectively and 92–100% in sham groups (Fig. 2E). Figure 2F–K demonstrate sample ECG recordings of various ventricular arrhythmias.

### ECG parameters and monophasic action potentials

To gain insight into mechanism of ventricular arrhythmias, we studied surface ECG parameters (continuous telemetric recording) and epicardial monophasic action potentials (MAPs).

**Figure 3:**
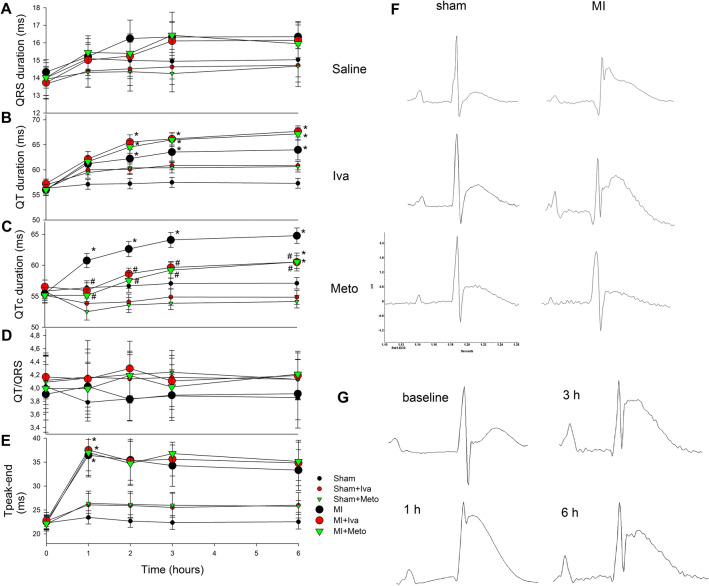
ECG parameters within 6 h of myocardial infarction induction or sham operation in rats (**A**) QRS duration, (**B**) QT duration, (**C**) corrected QT duration calculated using modified Bazett’s formula normalized to rat RR interval, (**D**) QT/QRS ratio, an index of cardiac electrophysiological balance, reflecting the wave length, (**E**) T_peak-end,_ interval from peak T wave to its end, reflecting duration of intramural dispersion of repolarization. (**F**) sample 2-lead ECG recordings from saline-, ivabradine and metoprolol-treated sham operated and MI rats, taken 6 h after sham operation/MI induction. (**G**) Evolution of MI in ECG recordings: baseline, 1 h, 3 h and 6 h after induction of MI. Data are means ± SEM. *p < 0.05 versus respective saline group; # *p* < 0.05 versus untreated MI. For group sizes see Table 1. Figure created using SigmaPlot v.14.0 (https://systatsoftware.com/products/sigmaplot/).

QRS duration did not change during the experiment: it was not affected either by MI induction or by any of the tested drugs (Fig. 3A). On the other hand, QT duration was increased 2 h after MI induction and tended to increase with time. Again, neither ivabradine nor metoprolol affected this phenomenon (Fig. 3B). However, QT interval corrected for HR using Bazett’s formula normalized to rat average RR duration, was affected by MI induction as early as 1 h after the MI induction and remained increased throughout the experiment (Fig. 3C). Moreover, both ivabradine and metoprolol were equally effective in preventing QTc prolongation induced by MI (Fig. 3C), while they did not affect QTc in sham operated rats (Fig. 3C). QT/QRS ratio, an index of cardiac electrophysiological balance, a marker of excitation wave length, another surrogate measure of predisposition to ventricular arrhythmia, was neither affected by MI or by any of the drugs (Fig. 3D). Last but not least, interval between peak amplitude of T wave and its end (T_peak-end_) was increased 1 h after MI induction and tended to decrease with time. Ivabradine and metoprolol had no effect on this parameter (Fig. 3E). Sample ECG signals from each group and MI evolution are presented in Fig. 3F,G, respectively.

**Figure 4:**
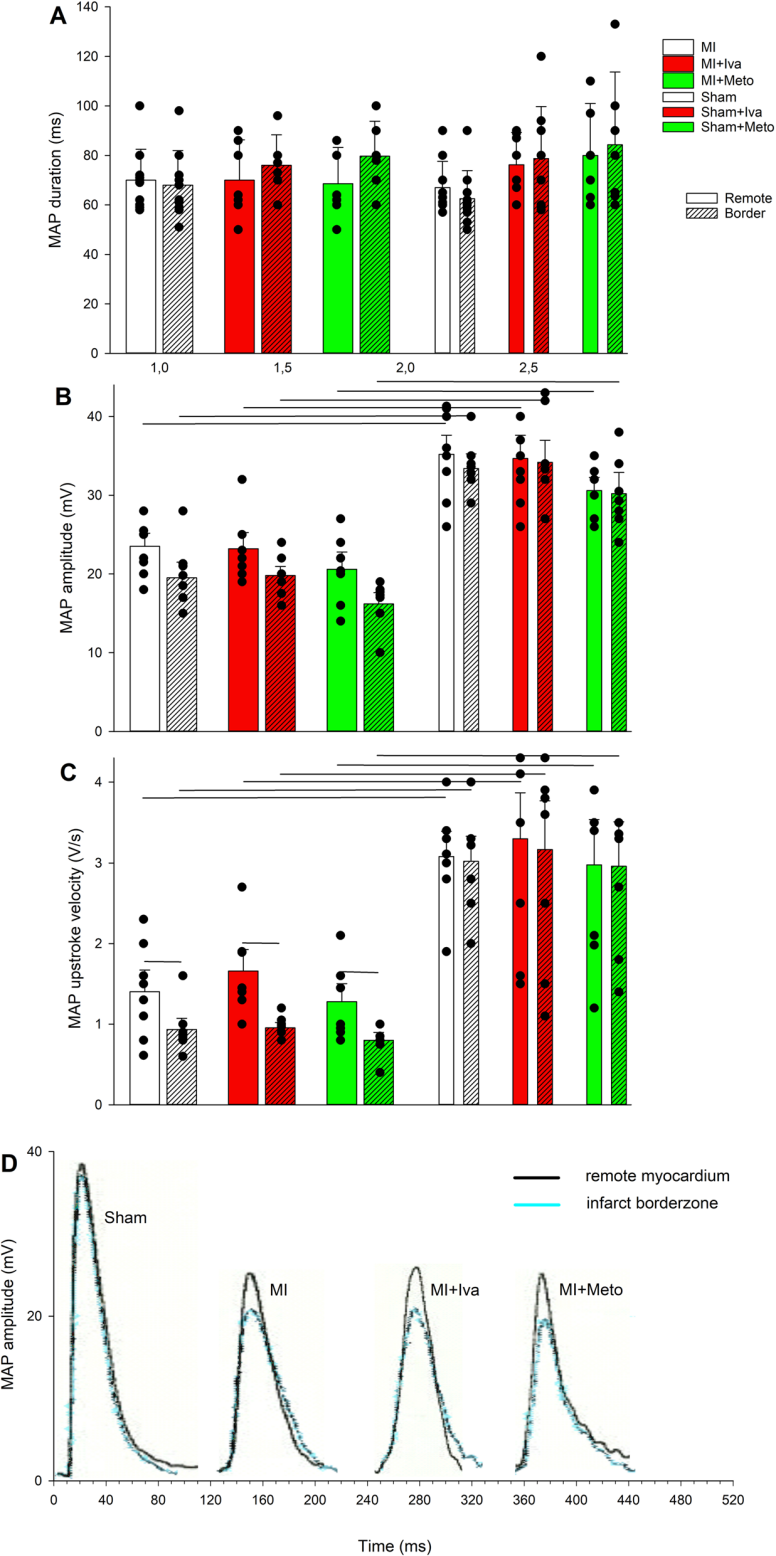
Epicardial monophasic action potentials (MAPs) recorded 6 h after myocardial infarction induction or sham operation in rats, separately in remote healthy left ventricular tissue (Remote) and infarct borderzone (Border) (**A**) MAP duration at 90% of action potential repolarization. (**B**) MAP amplitude. (**C**) MAP upstroke velocity. The solid circles represent the individual data points. (**D**) Sample MAPs from a sham operated animal, MI, MI + Iva and MI + Meto. Black line indicates MAP from remote LV tissue, blue one from infarct borderzone. The horizontal lines show statistical significance, *p* < 0.05. For group sizes see Table 1.

MAPs were recorded separately from epicardial surface of infarct borderzone and remote left ventricular (LV) tissue 6 h after MI induction/sham surgery. As Fig. 4A presents, MI induction had no effect on MAP duration either in the infarct borderzone or remote LV myocardium. Similarly neither ivabradine nor metoprolol affected MAP duration in sham or MI animals. (Fig. 4A). On the other hand, MAP amplitude and MAP upstroke velocity were reduced in all post-MI hearts. Ivabradine and metoprolol had no effect on these parameters (Fig. 4B and C). While we did not find any significant dispersion in MAP duration or amplitude between infarct borderzone and remote LV myocardium, MAP upstroke velocity was further impaired in the infarct borderzone versus remote LV tissue (Fig. 4C). Figure 4D presents sample MAP recordings from respective experimental groups.

### Cardiomyocyte calcium handling

We studied parameters of intracellular calcium handling in LV cardiomyocytes isolated from sham operated and MI rats 6 h after MI induction/sham surgery.

**Figure 5:**
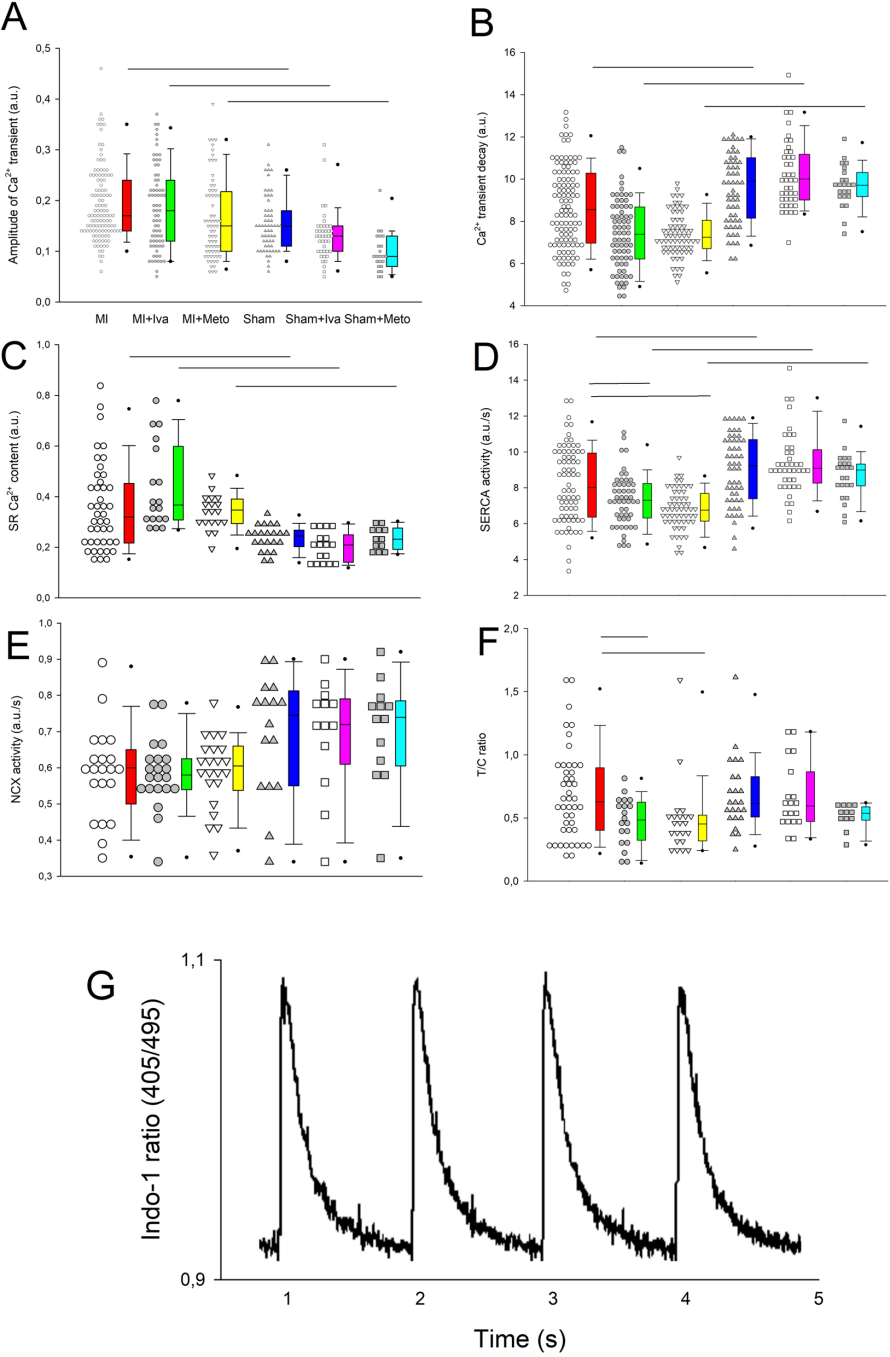
Ca^2+^ handling parameters in myocardial infarction or sham operated rats treated with saline, ivabradine or metoprolol (**A**) Amplitude of Ca^2+^ transients, (**B**) Ca^2+^ transient decay, (**C**) SR Ca^2+^ content, (**D**) sarcoplasmic Ca^2+^-ATPase (SERCA) activity, (**E**) sodium-calcium exchanger (NCX) activity, (**F**) amplitude of Ca^2+^ transient/SR Ca^2+^ content (T/C) ratio, (**G**) sample recording of electrically invoked Ca^2+^ transients. Mean ± SEM from (n = 42–118) cardiomyocytes isolated from at least 5 rats in each group. The horizontal lines show statistical significance, *p* < 0.05. a.u., arbitrary units. Figure created using SigmaPlot v.14.0 (https://systatsoftware.com/products/sigmaplot/).

Amplitude of Ca^2+^ transient and sarcoplasmic reticulum (SR) content were increased, while Ca^2+^ transient decay was reduced in post-MI cardiomyocytes (Fig. 5A–C). Ivabradine and metoprolol had no effect on these parameters of calcium handling either in Sham-operated or MI cardiomyocytes (Fig. 5A–C). Activity of SR calcium ATP-ase (SERCA), a principal enzyme responsible for diastolic SR filling, was impaired in post-MI cardiomyocytes (Fig. 5D) and ivabradine and metoprolol potentiated this effect to a similar degree. On the other hand, activity of plasma membrane sodium-calcium exchanger (NCX), an enzyme responsible for diastolic calcium extrusion from the cell, was neither affected by MI, nor by any of the tested drugs (Fig. 5E). Ca^2+^ transient/SR Ca^2+^ content ratio, a marker of ryanodine receptor sensitivity, was unchanged in post-MI cardiomyocytes, while both ivabradine and metoprolol reduced it (Fig. 5F).

## Discussion

Here we show that ivabradine, a putative selective HR reducing agent, reduces arrhythmic mortality and ventricular arrhythmias in the setting of acute myocardial infarction (MI) in the rat when given as oral gavage after MI induction. Its effects are comparable to those of metoprolol given at doses offering identical HR reduction. Possible beneficial effects of both these interventions seem to be related to HR reduction and apart from anti-ischemic effects include prevention of QT prolongation and reduction of sensitivity of ryanodine receptors. Furthermore, ivabradine does not raise any safety concerns in this setting, i.e. it does not exacerbate QT prolongation or dispersion of action potential duration within 6 h after MI induction.

Both arrhythmic mortality, arrhythmic incidence/duration and VPBs were particularly severe within 1 h after MI induction, though some mortality extended beyond this time frame, especially in untreated MI rats. Ivabradine and metoprolol markedly reduced these events within 1 h after MI induction and essentially abolished both arrhythmias and arrhythmic mortality after the 1 h mark, while reduction of VPBs was somewhat less impressive. Furthermore, both combined arrhythmia score, an index of the most severe form of arrhythmias, and rate of VT/VF free animals were significantly reduced by both ivabradine and metoprolol and to a similar degree.

Ventricular arrhythmias (ventricular tachycardia [VT] and ventricular fibrillation [VF]) arise in the post-MI setting mainly in the reentry mechanism. Initiation of reentry requires two factors: an arrhythmic trigger at a critical time and an arrhythmic substrate^15^.

Arrhythmic triggers involve ventricular premature beats (VPBs) that are induced by triggered activity or automaticity. Triggered activity can result from afterdepolarizations, i.e. voltage depolarizations (oscillations) that occur during the repolarization phase of the action potential (early afterdepolarizations, EAD) or during the diastolic phase, after completion of repolarization (delayed afterdepolarizations, DAD)^16^ (see Fig. 2F and G). If these voltage oscillations are large enough, they can trigger a full action potential, which is termed triggered activity. EADs are induced by reactivation of inward L-type calcium current, which is favored by prolongation of action potential duration. Alternatively spontaneous calcium release from the sarcoplasmic reticulum (SR) can activate sodium/calcium exchanger (NCX) generating inward current while exchanging one Ca^2+^ out for 3 Na^+^ ions in. DADs are induced by NCX activity and are favored by increased diastolic Ca^2+^ concentration and increased NCX activity. What is of note, EAD and DAD are quite similar, since both can occur under conditions of cardiomyocytes calcium overload and can be favored by spontaneous SR Ca^2+^ release and NCX overactivity^17^. Abnormally enhanced automaticity of working cardiac myocytes can be induced by increased extracellular K + concentration (resulting e.g. from ischemia), reduced expression/activity of potassium channels or increased expression/activity of ion channels responsible for inward depolarizing current (e.g., HCN4 channel, as we showed before in the post-MI setting^5^, although this mechanism is regarded as less important source of VEBs than afterdepolarizations.

To gain insight into mechanism of arrhythmias and potential antiarrhythmic activity of ivabradine and metoprolol we first tested intracellular calcium handling and ECG parameters. Factors that favor afterdepolarizations and eventually VPBs include: increased SR Ca^2+^ content (a marker of calcium overload), impaired SERCA activity, increased NCX activity and increased sensitivity of ryanodine receptors (RyR), responsible for SR Ca^2+^ release. Two out of these 4 factors were present in out post-MI cardiomyocytes: (1) increased SR Ca^2+^ content and (2) reduced SERCA activity. While neither ivabradine nor metoprolol affected SR Ca^2+^ content, they both further reduced SERCA activity, which should be regarded as a proarrhythmic effect. However, despite unchanged RyR sensitivity (as indicated by Ca^2+^ transient/SR Ca^2+^ content ratio [T/C] similar to that in sham cardiomyocytes), both ivabradine and metoprolol reduced it. It should be viewed as a beneficial influence on RyR sensitivity, an important marker of potential systolic and diastolic SR Ca^2+^ release favoring both EAD and DAD. Since our experiments were done with cells paced at constant frequency in all groups (1 Hz), they do not account for effects of HR reduction, which should further reduce SR Ca^2+^ content and related afterdepolarizations and arrhythmias^18^. However, corrected QT interval was increased after MI by almost 15%, possibly indicating increased risk of afterdepolarizations related to prolongation of action potential duration (as explained above). Both ivabradine and metoprolol were equally effective in alleviating this increase, which could at least partially account for their antiarrhythmic effects. Here we used QT corrected for HR using rat HR normalized Bazett’s formula that was previously shown to offer the best correction in the 300—400 bpm range of HR typical for the rat^19^. Summing up, we did not find evidence of any clear effects of either ivabradine or metoprolol on Ca^2+^ handling parameters that could explain their antiarrhythmic effects.

Myocardial activation depends on propagation of action potential and requires good cell–cell coupling and adequate cardiomyocytes excitability. Arrhythmic substrates involve factors that (1) slow the intracellular conduction and (2) increase the dispersion of repolarization. Slow intercellular conduction favors reentry, since it provides time for recovery of excitability of previously unexcitable tissue^15^. Intercellular conduction depends on two factors: amplitude and upstroke velocity of cardiomyocyte action potential (that depends on activation of sodium channels, SCN5A) and expression and function of gap junctions consisting of connexins that provide electrical connection between adjacent cells. Myocardial ischemia depresses cardiac conduction through partial depolarization of cardiomyocytes and impairment of activation of SCN5A, resulting in reduced amplitude and upstroke velocity of action potential; furthermore it uncouples gap junctions^20^, which contributes to ventricular arrhythmias in the acute MI setting.

We used two approaches to estimate intercellular conduction. First, QRS duration is a known global marker of total duration of LV depolarization and hence its dynamic changes are an indirect marker of changes of the intercellular conduction velocity. In our study QRS did not change significantly, although there was a clear trend toward its mild increase with time after MI induction. Neither drug intervention affected this. As another approach, we used epicardial monophasic action potentials (MAP) recording. MAPs are wave forms of transmembrane action potential recorded from the epicardium that can be obtained from beating hearts in situ. However, the difference between MAP and transmembrane action potential (TAP) is that while TAP is a recording of transmembrane potential of a single cell and thus can be obtained only in vitro, MAP is a measure of a small group of cells < 5 mm in diameter^21^ and therefore it reflects “averaged” action potentials of this group of cells. Thus MAP upstroke velocity is ~ 2 orders of magnitude lower than that of TAP, though its changes reliably reflect changes of TAP upstroke velocity^21^.

MAP amplitude and upstroke velocity were markedly depressed in all post-MI groups, both in the infarct borderzone and in LV remote myocardium. While changes in the infarct borderzone could be explained by ongoing ischemia, depression of these parameters in the LV remote myocardium, not subjected to an ischemic insult, might be surprising. However, we^22^ and others^23^ have shown that LV wall stress is increased immediately after MI induction and abnormally elevated wall stress has been shown to affect both calcium handling^24^ and electrophysiological properties of the myocardium, including depression of conduction velocity, probably due to activation of stretch-activated ion channels and partial depolarization of cardiomyocytes^25^. This could explain reduction of MAP amplitude and upstroke velocity in LV remote myocardium and thus increased propensity to ventricular arrhythmias.

QRS duration, a global marker of LV conduction, did not change despite changes in MAP upstroke and amplitude. One explanation for this discrepancy is that the LV conduction reserve is quite large, allowing for marked reduction of determinants of intercellular conduction before it affects global conduction. One study reported a French family with heterozygotes carried an inactive allele of SCN5A^26^, resulting in 50% functional Na channels. Young subjects presented with normal QRS duration in ECG. QRS duration started to increase only after the age of 40. This indicates that the LV conduction reserve is large and additional factor, such as aging, may be required. Nevertheless, neither intervention used in our study affected any of MAP parameters.

Another proarrhythmic substrate favoring ventricular arrhythmias is dispersion of LV repolarization related to dispersion of action potential duration and conduction since it creates favorable conditions for reentry via unidirectional conduction block. Heterogeneous action potential duration, both transmural (across the myocardial wall) and transventricular (across the ventricles of the heart, mainly reflecting differences between the healthy LV remote tissue and the infarct borderzone) as well as heterogeneous conduction between the LV remote and borderzone tissue are the main determinants of this dispersion. Again we used two strategies to study dispersion of LV repolarization: surface ECG and MAPs. T_peak-end_, a marker of transmural dipersion of repolarization, was recently shown to be increased in acute phase of MI and predict ventricular arrhythmias within 24 h of MI^27^. Indeed, we found T_peak-end_ to be increased 1 h after MI induction and then tend to decrease with time. However, neither ivabradine nor metoprolol affected it. We did not find dispersion of MAP duration or amplitude between LV remote and borderzone, however MAP upstroke velocity was significantly depressed in LV borderzone, potentially contributing to favorable conditions to reentry formation. However, again, neither of our interventions had any effect on this parameter. Thus prevention of dispersion of LV repolarization is unlikely to contribute to ivabradine or metoprolol antiarrhythmic effects in our study.

Recently a new index of propensity to reentry arrhythmias was developed—an index of cardiac electrophysiological balance iCEB^28^, QT interval/QRS duration. The excitation wavelength given by conduction velocity times effective refractory period is the minimum path length that can support a reentrant circuit and represents the link between cellular depolarization and repolarization^29^. It can be approximated by the ratio of the duration of repolarization and depolarization, i.e., QT interval/QRS duration^28^ and it can be easily derived from ECG. In our study this index was neither affected by MI, not by any interventions.

We used metoprolol and ivabradine at doses that offered immediate (within 10 min of administration) and nearly identical HR reduction that persisted throughout the experiment, up to 6 h after induction of MI. Thus we were able to test if either of these drugs provided any additional effects extending beyond HR reduction. Lack of specific electrophysiological effects of ivabradine and similar efficacy to that of metoprolol strongly suggest that antiarrhythmic activity of ivabradine is related to its HR reducing ability. Indeed, Ng et al.^30^ previously demonstrated that atrial pacing that prevented HR reduction abolished antiarrhythmic effects of ivabradine in isolated rat hearts subjected to ischemia and reperfusion. They showed that HR during ischemia was a determinant of arrhythmogenesis and that reduced HR delayed the onset of loss of electrical excitability. In another study VF threshold in pigs subjected to ischemia and reperfusion was reduced by ivabradine and this reduction was related to HR reduction^31^. To further elucidate if potential anti-ischemic effects of ivabradine and metoprolol count be responsible for their antiarrhythmic effects we compared the time to peak ST elevation (as a marker of the rate of ischemia development) and time to Q wave development (as a marker of the rate of necrosis development), but found no differences among the MI groups, making them unlikely to contribute to the observed effects.

Ivabradine was clearly antiarrhythmic in our post-MI rats, reducing arrhythmic mortality, incidence and duration of ventricular arrhythmias as well as VPBs to a similar degree as an established therapy, metoprolol. Furthermore it did not prolong QT or MAP as might be expected had it inhibited *I*_Kr_ and even more, ivabradine actually markedly reduced prolongation of QTc in post-MI rat hearts. Moreover, ivabradine did not increase markers of either transmural or transventricular dispersion of repolarization. Thus in this model of non-reperfused myocardial infarction in the rat there is no evidence of any potential proarrhythmic effects of ivabradine after its acute administration.

Of note, electrophysiology of the rat differs from that in humans in that a more prominent *I*_to_ is responsible for much shorter action potential duration, while *I*_Kr_ is less prominent than in humans, therefore potential effects of *I*_Kr_ inhibition may be underestimated in our model. This is a limitation of our study, reducing translational value of our work. However, *I*_Kr_ has been shown to be functionally downregulated in human heart failure, which drastically reduces its contribution to repolarization^32^. Therefore, regarding repolarization, the rat may be viewed as a model resembling human heart failure. On the other hand, the other phases of action potential are very similar in rodents as in humans and fundamental mechanisms of arrhythmias are essentially identical^17^. Moreover, we did not compare antiarrhythmic effects of ivabradine to that of conventional antiarrhythmic agents such as amiodarone, since the latter are not approved for the treatment of patients with acute myocardial infarction and are not routinely used in this setting.

In conclusion, we show that ivabradine is as effective in reducing ventricular arrhythmias in the non-reperfused myocardial infarction in the rat as metoprolol when both drugs are given at doses providing the same HR reduction. Furthermore ivabradine does not cause any proarrhythmic effects that could be attributable to its putative *I*_Kr_ inhibition. Lack of specific electrophysiological effects and similar efficacy to that of metoprolol strongly suggests that antiarrhythmic activity of ivabradine is related to its HR reducing ability. Ivabradine could potentially be a viable alternative to ß-blockers as an antiarrhythmic treatment devoid of hemodynamic effects when ß-blockers are contraindicated.

## Methods

One hundred and eighty male Wistar–Kyoto rats, weighing 270–310 g, were used. All study animals were used in compliance with local and institutional regulations. The study conformed to the Guide for the Care and Use of Laboratory Animals, US National Institutes of Health (NIH Publication No. 85–23, revised 1996) and was approved by the local ethics committee (Second Warsaw Local Ethics Committee for Animal Experimentation).

### Preliminary experiments: ivabradine and metoprolol administration

**Figure 6:**
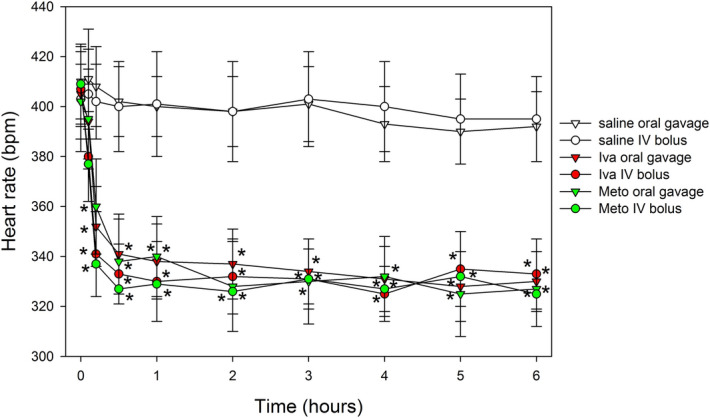
Time course and magnitude of heart rate reduction following administration of ivabradine and metoprolol as intravenous bolus or oral gavage. *p < 0.05 versus respective saline group. Each group n = 5 animals. Figure created using SigmaPlot v.14.0 (https://systatsoftware.com/products/sigmaplot/).

A preliminary set of experiments was performed to compare effects of ivabradine and metoprolol administration, by oral gavage and intravenous, on HR (ivabradine 5 mg/kg and 2 mg/kg, metoprolol 20 mg/kg and 5 mg/kg, respectively). Figure 6 demonstrates that both methods of administration provided effective, rapid and equipotent HR reduction that persisted for at least 6 h (duration of the proper experiment). Since the effects were comparable, we decided to use oral gavage as a method of ivabradine and metoprolol administration in the proper experiments. Ivabradine was kindly provided by Servier Laboratories, France and metoprolol by Polpharma, Poland.

### Study protocol

The rats were anesthetized (10% chloral hydrate, 3 ml/kg body weight; intraperitoneal; to ensure proper analgesia, acetaminophen [67 mg/ml drinking water] was given for 4 h before and until the end of the experiment; chloral hydrate was used to avoid effect of anesthesia on either HR or arrhythmia). Then the telemetry transmitter was implanted, MI induction or sham operation was performed and ivabradine (5 mg/kg), metoprolol (20 mg/kg) or saline were given as oral gavage within 1 min of MI induction/sham operation. Thus the following 6 groups were obtained: sham + saline (n = 14), sham + ivabradine (n = 15), sham + metoprolol (n = 14), MI + saline (n = 41), MI + ivabradine (n = 33) and MI + metoprolol (n = 33). Ten minutes later echocardiography was used to confirm moderate or large MI (rats with small MI were excluded from the study, a total of 13 animals). Six hours after the surgery the rats were anesthetized again, epicardial monophasic action potentials (MAPs) were recorded and hearts were processed for cardiomyocytes isolation.

### Implantation of telemetry transmitter and induction of myocardial infarction

A continuous ECG telemetry transmitter (Data Sciences International, St. Paul,MN) was implanted under the skin of the abdomen under anesthesia. The positive electrode was placed in a V_5_ position, the negative electrode under the right axilla and the rat was placed on the receiver that continuously captured the signal. The ECG signal was recorded (A.R.T. 4.0, Data Sciences International) until the end of the experiment. One hour later, after good quality and stable ECG recording was confirmed, left thoracotomy was performed. The heart was externalized and a suture was placed around the proximal left coronary artery and tightly tied to induce MI or was not tied (sham operated rats), as we reported previously^33^. The heart was internalized and the chest was closed. No attempt was made to resuscitate animals with ventricular arrhythmias.

Echocardiography was performed using MyLab25 (Esaote, Italy) with 13 MHz linear array transducer. Regional LV wall motion abnormalities were quantitated using the wall motion index (WMI). We have previously shown that WMI is a good echocardiographic indicator of infarct size in the rat^34^. Rats with WMI < 18 were considered to have moderate or large MI (WMI = 18 corresponds to an infarct involving ~ 28% of the LV myocardium).

### ECG and arrhythmia analysis

The acquired single-lead ECG tracings were analyzed manually off-line. Ventricular arrhythmias were classified according to the Lambeth Conventions guidelines, as reported previously^5^. Ventricular tachycardia (VT) was defined as 4 or more consecutive ventricular premature beats with an average frequency of at least 500 bpm. VF was defined as a signal that changed from beat to beat in rate and morphology or a signal in which individual QRS deflections could not easily be distinguished from one another. As reported previously^35^, since separating VF from VT was often difficult, they were tabulated together. In the analysis of arrhythmia duration, each case of fatal VF was assigned a duration of 120 s (the longest observed nonfatal VF episode). Heart rate was calculated from ECG recordings from which nonsinus beats were excluded. Combined arrhythmia score was calculated as described previously^36^, with modifications. Briefly, each rat was assigned a score corresponding to the most severe ventricular arrhythmia it experienced, where 0 corresponded to no arrhythmia or no more than 10 VPBs; 1—more than 10 VPBs; 2—double or triple VPBs; 3—monomorphic VT, duration < 15 s; 4—monomorphic VT, duration ≥ 15 s; 5—polymorphic VT or VF; 6—VT or VF resulting in death. Corrected QT (QTc) normalized to rat average RR duration was calculated according to the following formula: QTc = QT/[(RR interval/150)^1/2^] [ms].

### Monophasic action potentials

The local subepicardial monophasic action potentials (MAPs) were recorded using a miniature suction electrode consisting of silver wire (∅ = 0.5 mm) protruding from the small plastic tube connected to the vacuum source, as reported previously^5^. The reference electrode was attached to the outer surface of the plastic tube. To record MAPs, the surface of the heart was gently touched with the measuring electrode and suction was applied. MAPs were recorded from two points of the free non-infarcted (remote) LV wall: near the base and halfway between the base and the apex, and from the two points in the infarct borderzone (a 2-mm zone surrounding the infarct). Five consecutive MAPs were averaged for each point and subsequently averaged from two points in each area. In the sham operated rats MAPs were recorded from the corresponding points.

### Myocyte isolation, Ca^2+^transient recording, rate of Ca^2+^transport by SERCA, NCX and sarcoplasmic reticulum Ca^2+^ content

The LV myocytes were isolated by enzymatic digestion, as described previously^33^, and superfused at 37 °C with Tyrode’s solution containing 1.8 mmol/l Ca^2+^. Ca^2+^ transient was recorded using indo-1 fluorescence (excited at 365 and measured at 405 and 495 nm; for a sample recording see Fig. 5G). The rate of Ca^2+^ transport by sarcoplasmic reticulum (SR) Ca^2+^-ATPase (SERCA) and Na^+^/Ca^2+^ exchanger (NCX) and plasma membrane Ca^2+^-ATPase (PMCA) was estimated from the rate constants (r1, r2, r3) of the single exponential curves fitted to electrically- and caffeine-evoked Ca^2+^ transients decay. The rate constants of the Ca^2+^ transient decay for SERCA and NCX was calculated according to formulas: r_SERCA_ = r1-r2 and r_NCX_ = r2-r3, respectively, while r3 was taken as the measure of the rate of Ca^2+^ transport by PMCA (r3 = r_PMCA_) (see details^33^). r_SERCA_, r_NCX_ and r_PMCA_ describe average velocity of Ca^2+^ transport by SERCA, NCX and PMCA, respectively. SR Ca^2+^ content was estimated from the amplitude of caffeine-evoked Ca^2+^ transients in myocytes superfused with Na^+^, Ca^2+^-free (0Na0Ca) solution.

### Statistical analysis

Normality of data distribution was tested using the Shapiro–Wilk test, while homogeneity of variances by Bartlett’s test. Normally distributed data were expressed as means ± SEM. Differences between groups were tested by one-way ANOVA. Two-way repeated-measures ANOVA was used to compare time-dependent measurements. Tukey post hoc test was used to compare data pairs. If data were not normally distributed, they were presented as median ± inter-quartile ranges + outliers, if applicable. Statistical analysis of differences was then made using non-parametric methods (Mann–Whitney test to compare two groups, or Kruskal–Wallis ANOVA to compare three or more groups followed by Dunn's post hoc test). Differences were considered significant when *p* < 0.05. Statistical analyses were performed using SigmaPlot v. 11.

## Data Availability

All data are available at the Department of Clinical Physiology, Centre of Postgraduate Medical Education, Warsaw, Poland.
